# Frequent occurrence of mutations in nsp3 and nsp4 of SARS-CoV-2, presumably caused by the inhaled asthma drug ciclesonide

**DOI:** 10.1093/pnasnexus/pgac197

**Published:** 2022-09-20

**Authors:** Akihiro Doi, Yuriko Tomita, Hiyori Okura, Shutoku Matsuyama

**Affiliations:** Research Center for Influenza and Respiratory Viruses, National Institute of Infectious Diseases, Murayama Branch, 4-7-1 Gakuen, Musashi-Murayama, Tokyo 208-0011, Japan; Research Center for Influenza and Respiratory Viruses, National Institute of Infectious Diseases, Murayama Branch, 4-7-1 Gakuen, Musashi-Murayama, Tokyo 208-0011, Japan; Research Center for Influenza and Respiratory Viruses, National Institute of Infectious Diseases, Murayama Branch, 4-7-1 Gakuen, Musashi-Murayama, Tokyo 208-0011, Japan; Research Center for Influenza and Respiratory Viruses, National Institute of Infectious Diseases, Murayama Branch, 4-7-1 Gakuen, Musashi-Murayama, Tokyo 208-0011, Japan

**Keywords:** SARS-CoV-2, asthma, ciclesonide, GISAID

## Abstract

Mutations in nonstructural protein 3 (nsp3) and nsp4 of SARS-CoV-2, presumably induced by the asthma drug ciclesonide (which also has anti-SARS-CoV-2 activity), were counted 5,851 cases in the GISAID EpiCoV genome database. Sporadic occurrence of mutants not linked to each other in the phylogenetic tree were identified at least 88 times; of which, 58 had one or more descendants in the same branch. Five of these had spread to more than 100 cases, and one had expanded to 4,748 cases, suggesting the mutations are frequent, selected in individual patients, and transmitted to form clusters of cases. Clinical trials of ciclesonide as a treatment for COVID-19 are the presumed cause of the frequent occurrence of mutations between 2020 June and 2021 November. In addition, because ciclesonide is a common treatment for asthma, it can drive mutations in asthmatics suffering from COVID-19. Ciclesonide-resistant mutations, which have unpredictable effects in humans, are likely to continue to emerge because SARS-CoV-2 remains prevalent globally.

Significance statement The inhaled asthma drug ciclesonide suppresses SARS-CoV-2 replication. In our laboratory, exposure of SARS-CoV-2-infected cultured cells to ciclesonide led to emergence of ciclesonide-resistant mutants. These experimentally-induced mutations were predicted to also occur in asthmatics with COVID-19 because they inhale ciclesonide. In this study, we searched for ciclesonide-resistant mutations in the GISAID EpiCoV database and surprisingly found 88 sporadic occurrences, of which 58 mutants had one or more descendants in the same branch of the phylogenetic tree, suggesting that the mutations occurred frequently and were transmitted to other people.

## Introduction

Next generation sequencing (NGS) has produced a huge number of SARS-CoV-2 genome sequences (1). SARS-CoV-2 emerged at the end of 2019, and by 2022 March, the number of sequences registered in the GISAID EpiCoV database reached 9,896,001. It is now possible to trace the transmission of viruses on a phylogenetic tree and to recognize new variants as soon as they emerge.

Ciclesonide, an inhaled corticosteroid, is a safe anti-inflammatory drug used for the long-term treatment of asthma and allergic rhinitis (2). Previously, our group and others reported that ciclesonide is a potent blocker of SARS-CoV-2 replication (3, 4). This finding led to clinical trials of ciclesonide as a COVID-19 therapy in some countries. However, these trials failed to show a clear therapeutic effect (5–7). On the other hand, we isolated ciclesonide-resistant mutants from cell cultures after eighth passages of SARS-CoV-2 in the presence of 40 µM ciclesonide (4). The amino acid substitutions in nsp3 and nsp4 of SARS-CoV-2 were identified, suggesting that ciclesonide interacts with the replication-transcription complex which constructed of nsp3 and nsp4 in virus infected cells, and inhibits viral RNA replication (4).

In this study, we searched for ciclesonide-resistant mutations in the GISAID EpiCoV database and confirmed that mutations induced experimentally in the laboratory occurred in specimens collected from patients.

## Results

**Fig. 1.**
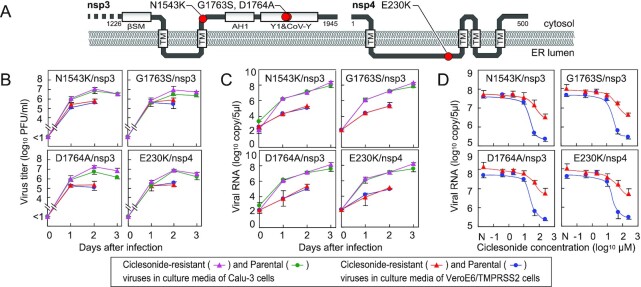
Replication of ciclesonide-resistant viruses in cultured cells. (A) Membrane topology of the nsp3 C-terminal domain and nsp4 showing the locations of ciclesonide-resistant mutations. (B) Time course of virus replication. (C) Quantification of viral RNA in the culture medium used in panel B. (D) Concentration-dependent effects of ciclesonide on virus replication. N, no drug treatment.

First, we examined the replication and the drug-resistance potential of ciclesonide-resistant viruses harboring a single mutation in nsp3 or nsp4 (Fig. 1A). Human bronchial epithelial Calu-3 cells and VeroE6/TMPRSS2 cells were infected with two clones of each mutant (i.e. eight independent clones), along with their parental viruses, and the amount of virus secreted into the culture medium was quantified by the plaque assay (Fig. 1B) or real-time PCR (Fig. 1C). The mutants grew similarly to the parental viruses. In the presence of 50 μM ciclesonide, replication of ciclesonide-resistant mutants was 30-fold higher than that of the parental viruses (Fig. 1D). These observations suggested that mutations in nsp3 or nsp4 are involved in ciclesonide resistance and confer a replication advantage in the presence of ciclesonide.

Next, we searched the GISAID EpiCoV database, which contains viral genome sequences collected from COVID-19 patients, for SARS-CoV-2 mutations causing ciclesonide resistance. For comparison, a mutation E802D/nsp12, involved in resistance to the antivirus drug remdesivir, was employed (8). Synonymous nucleotide substitutions of U7348A of U7348G for N1543K/nsp3, and G15846C of G15846U for E802D/nsp12 were also collected from the database. In addition, mutations unlikely to be introduced by any drug, namely the D1764E/nsp3 substitution at the same site in the mutant that causes ciclesonide resistance (i.e. D1764A/nsp3) and the Q498R/spike mutation identified in Omicron variants (9) were also employed. The whole genome sequences of these mutants, registered in the database between 2020 January and 2022 March were downloaded and phylogenetic trees were constructed using the Nextstrain interface.

**Fig. 2.**
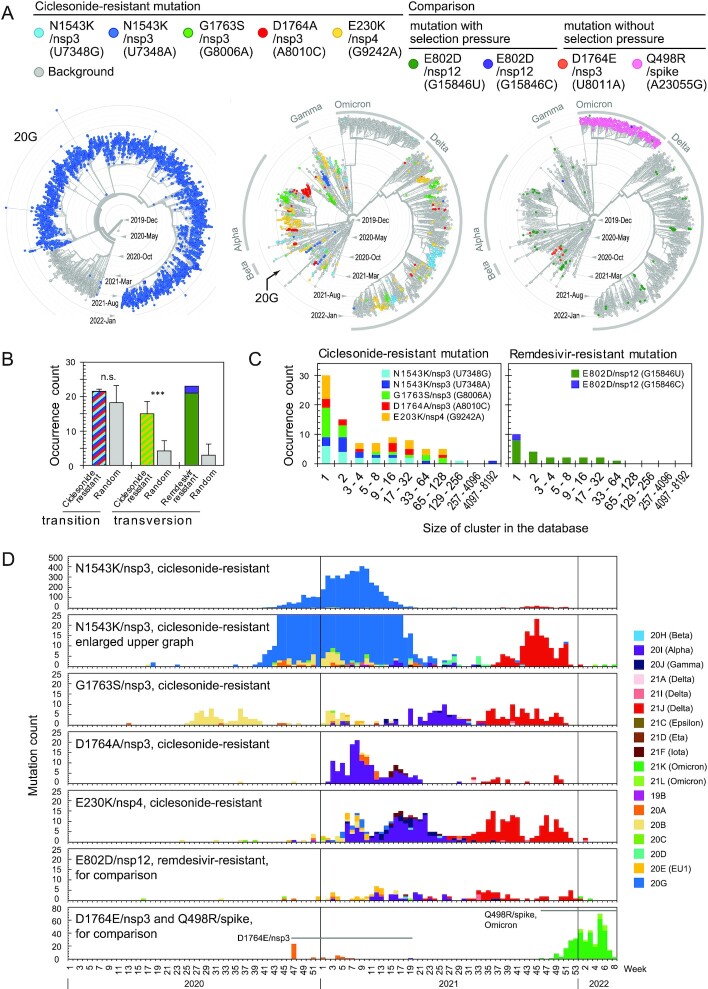
Ciclesonide-resistant mutants in the GISAID EpiCoV genome database. (A) Phylogenetic trees. (B) Statistical analysis of mutant occurrence. Colors correspond to the mutations in panel A. n.s., not significant and ***, significant difference (*P* < 0.001). (C) Occurrence number of mutants according to cluster size. (D) Weekly count of cases.

Ciclesonide-resistant mutations are indicated in the phylogenetic tree (Fig. 2A, center). The number of mutations was as follows: N1543K/nsp3 (U7348G), 211; N1543K/nsp3 (U7348A), 4816; G1763S/nsp3,260; D1764A/nsp3,180; and E230K/nsp4,384; i.e. 5,851 in total. One mutant in clade 20 G harboring N1543K/nsp3 (U7348A) had expanded to form a large cluster of 4,748 cases due to an outbreak in the United States, which made constructing the phylogenetic tree awkward. Therefore, the 20 G clade is shown separately in the left panel of Fig. 2A. No other geographical-related occurrences of mutants were observed. Sporadic occurrences of mutants not linked to each other in the phylogenetic tree were counted using the following rule: if only one case occurred in a clade, or one case occurred at least 2 segments downstream of different branches from other cases belonging to the same clade, this was counted as a single occurrence. The number of sporadic occurrences of mutants was as follows: N1543K/nsp3 (U7348G), 19 times; N1543K/nsp3 (U7348A), 14 times; G1763S/nsp3, 21 times; D1764/nsp3, 12 times; and E230K/nsp4, 22 times; i.e. 88 times in total. The occurrence of these mutations was statistically compared to that of random mutations as described in Supplementary Material. A slightly higher number of transitions (G8006A and G9242A) was observed above the high background level of natural mutations, whereas the number of transversions (U7348G, U7348A, and A8010C) were significantly higher than that of random mutations. A similar difference was observed between the remdesivir-resistant mutation and the random mutations (Fig. 2B). With respect to cluster size, a single case occurred 30 times, more than one case occurred 58 times, more than eight cases occurred 29 times, and more than 100 cases occurred five times (Fig. 2C). These observations suggest that the ciclesonide-resistant mutant is common, becomes dominant in each patient, and then is transmitted to form clusters of cases. Most mutants were identified between 2020 June and 2021 November (Fig. 2D), which corresponds roughly with a period of worldwide clinical trials of ciclesonide as a treatment for COVID-19 enrolled at the following web sites: clinicaltrials.gov/, eudract.ema.europa.eu/, anzctr.org.au/, jrct.niph.go.jp/, and ctri.nic.in/. By comparison, the sporadic occurrence of the remdesivir-resistant mutant E802D/nsp12 (G15846U) was 21 times, and it had spread to a total of 112 cases in the database. Most remdesivir-resistant mutants formed smaller clusters than those of ciclesonide-resistant mutants (Fig. 2C). In addition, the D1764E/nsp3 and the Q498R/spike mutations counted for only three and one occurrences, respectively. The mutants resistant to ciclesonide or remdesivir can be thought to replicate predominantly under drug selection pressure, resulting in their occurrence, but the D1764E/nsp3 and the Q498R/spike mutations occurred in the absence of selection pressure.

## Discussion

Ciclesonide is an approved anti-asthma drug with a large market in South America, North America, and the Asia-Pacific (10, 11). Because ciclesonide has few side effects in humans, it is administered at high concentrations (5, 7). We expect that asthmatics infected with SARS-CoV-2 will use ciclesonide because COVID-19 and asthma have similar respiratory symptoms including wheezing, shortness of breath, and cough (12, 13). Furthermore, ciclesonide is commonly self-administered by nonhospitalized asthma patients, increasing the chances that a resistant mutant will emerge and spread. Ciclesonide inhaled in powder form at 400 μg/day for asthmatics dissolves in the small amount of exudate that covers the surface of the respiratory tract, presumably delivered a sufficient concentration to allow the emergence of resistant viruses, as described in the previous study (4). Thus, ciclesonide exerts high selection pressure on the virus in COVID-19 patients with asthma, thereby presumably driving emergence of resistant mutants.

## Materials and Methods

See the extended “Methods” section in the Supplementary Material for full details of the methods and data analysis strategies discussed in this paper.

## Supplementary Material

pgac197_Supplemental_File

## Data Availability

All data are included in the manuscript and/or supporting information.

## Notes

A.D. and Y.T. contributed equally to this work.
