# Supplementary material for: Frequent occurrence of mutations in nsp3 and nsp4 of SARS-CoV-2, presumably caused by the inhaled asthma drug ciclesonide
Source: PNAS Nexus. 2022 Sep 20;1(4):pgac197. doi: 10.1093/pnasnexus/pgac197 (PMC9802299; doi:10.1093/pnasnexus/pgac197)
Supplement: pgac197_Supplemental_File [file pgac197_supplemental_file.docx]

**
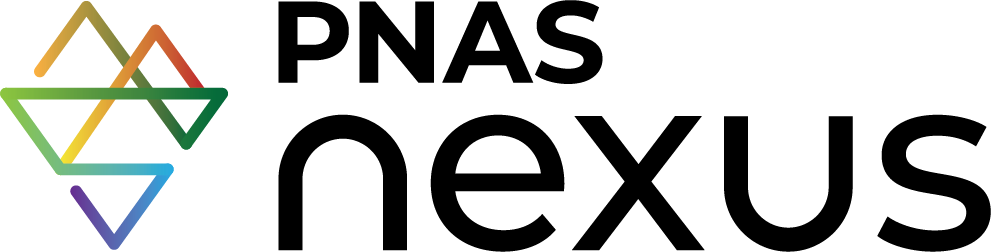
**

**Supplementary Information for**

Frequent occurrence of mutations in nsp3 and nsp4 of SARS-CoV-2, presumably caused by the inhaled asthma drug ciclesonide

Akihiro Doi, Yuriko Tomita, Hiyori Okura, and Shutoku Matsuyama

Shutoku Matsuyama

Email: matuyama@niid.go.jp.

**This PDF file includes:**

Materials and Methods

SI References

**Supplementary Information Text**

**Materials**

**Viruses**

Eight ciclesonide-resistant SARS-CoV-2 mutants, each harboring a single mutation in nsp3 or nsp4, were isolated from their parental viruses in our laboratory (1). Briefly, 43 independent SARS-CoV-2 isolates in our laboratory from infected patients were consecutively passaged in VeroE6/TMPRSS2 cells in the presence of 40 µM ciclesonide. After eight passages, three viral plaques from each passage were isolated using limiting dilution. The viruses were propagated in VeroE6/TMPRSS2 cells (2), and the viral RNA was then isolated for next-generation sequencing. Full genome sequences were compared with the reference sequence of the original strain that emerged in 2019 (GenBank ID: MN908947). We obtained 15 isolates harboring a single mutation and 22 isolates harboring multiple mutations that were absent from the parental strain (1). The parental viruses were named DP15-200, DP16-090, DP16-074, DP17-187, DP16-078, DP17-243, DP15-104, and DP16-238. The respective mutants were named N1543K/nsp3-clone-1 and -2, G1763S/nsp3-clone-1 and -2, D1764A/nsp3-clone-1 and -2, and E230K/nsp4-clone-1 and -2.

**Methods**

**Quantification of viral replication**

Human bronchial epithelial Calu-3 cells and VeroE6/TMPRSS2 cells cultured in a 96-well plate were inoculated with SARS-CoV-2 at an MOI of 0.01 and 0.001, respectively. After incubation for up to 3 days, culture media were collected and the virus titer was measured by a plaque assay using VeroE6/TMPRSS2 cells. For real-time PCR assays, medium was diluted 10-fold in water, and then boiled for 10 minutes. The following primers and probe were used: the primes were E_Sarbeco_F, ACAGGTACGTTAATAGTTAATAGCGT, E_Sarbeco_R, ATATTGCAGCAGTACGCACACA, and the probe was E_Sarbeco_P1-FAM, ACACTAGCCATCCTTACTGCGCTTCG, as described previously (3). Real-time PCR was performed using a LightCycler480 System II apparatus (Roche, Basel, Switzerland).

**Quantification of viral replication inhibition**

The cells described above were inoculated with SARS-CoV-2 at an MOI of 0.01 in the presence of DMSO (vehicle control) or various concentrations of ciclesonide. After 24 hours incubation, culture media were collected and the viral RNA was quantified by real-time PCR as described above.

**Phylogenetic tree**

Phylogenetic tree construction and maximum likelihood (ML) analysis were performed using the workflow provided by the Nextstrain command-line interface (CLI) (4). First, SARS-CoV-2 full-genome sequences harboring ciclesonide- and remdesivir-resistant mutations, respectively, were downloaded from the GISAID EpiCoV database (https://www.epicov.org/). Low quality NGS data were excluded prior to download. To draw the phylogenetic tree covering all SARS-CoV-2 variants, 1,989 background genomes were obtained from Nextstrain (https://nextstrain.org/ncov/gisaid/global/all-time) registered between December 26th, 2019 and February 23th, 2022. The 993 genome sequences for clade 20G, all of which harbored the nsp3 M1788I mutation specific to clade 20G, were also included as a background for the nsp3 N1543K (U7348A) mutant. Next, SARS-CoV-2 genomes were aligned using MAFFT version 7, (https://mafft.cbrc.jp/alignment/server/add_sarscov2.html), followed by trimming of the 5’ and 3’ UTRs using GENETYX-MAC Ver.21 (GENETYX CORPORATION, Tokyo, Japan). The MAFFT aligned genomes were subjected to “ncov” workflow pipeline analysis (https://github.com/nextstrain/ncov). Phylogenetic trees were visualized on auspice.us (https://auspice.us/).

**Statistical analysis of mutant occurrence**

The occurrence of drug-induced mutation was statistically compared with the natural occurrence of random mutations. The ciclesonide-resistant mutation N1543K changed the nucleotide triplet AAU coding for asparagine to AAA or AAG coding for lysine; these coding changes were observed 14 and 19 times, respectively, in the phylogenetic tree. The counting rules of mutant occurrence are described in the main text. The random mutations naturally occurring at amino acid position 1543 were AAC, AUU, AGU, ACU, UAU, GAU, and CAU. Of note, in SARS-CoV-2 mutagenesis, transitions were reported to be 3.3-fold more frequent than transversions (5); therefore, the transitions and the transversions were analysed separately. Because the mutation N1543K is caused by a transversion, U>G or U>A, four transversions were chosen from random mutations and their occurrences in the phylogenetic tree were AUU, 3; ACU, 9; UAU, 4; and CAU, 1. The occurrences of the other three ciclesonide-resistant mutations, G1763S/nsp3, D1764A/nsp3, and E230K/nsp4, and the remdesivir resistant mutation, E802D/nsp12, were also counted, and a student’s t-test was carried out to clarify the significant difference between the drug-induced mutations and the random mutations.

**SI References**

1. S. Matsuyama, *et al.*, The inhaled steroid ciclesonide blocks SARS-CoV-2 RNA replication by targeting the viral replication-transcription complex in cultured cells. *J. Virol.* **95**, e01648-20 (2020).

2. S. Matsuyama, *et al.*, Enhanced isolation of SARS-CoV-2 by TMPRSS2- expressing cells. *Proc. Natl. Acad. Sci. U. S. A.* **117**, 7001–7003 (2020).

3. V. M. Corman, *et al.*, Detection of 2019 novel coronavirus (2019-nCoV) by real-time RT-PCR. *Eurosurveillance* **25**, 2000045 (2020).

4. J. E. Lemieux, *et al.*, Phylogenetic analysis of SARS-CoV-2 in Boston highlights the impact of superspreading events. *Science* **371**, eabe3261 (2021).

5. C. Roy, *et al.*, Trends of mutation accumulation across global SARS-CoV-2 genomes: Implications for the evolution of the novel coronavirus. *Genomics* **112**, 5331–5342 (2020).
